# Specific genomic alterations and aggressive clinical features of sporadic thyroid carcinomas in children and adolescents: findings from an in-house cohort study

**DOI:** 10.3389/fendo.2025.1603571

**Published:** 2025-08-15

**Authors:** Gongxun Tan, Yuguo Wang, Guoliang Zhang, Xian Wang, Yongzhen Ren, Qian Gu, Feiju Xu, Zhenwei Mao, Chunhe Shi, Hui Wang, Ting Wu, Xi Wei, Tengxu Zhang, Xiuying Li, Ying Xu, Shengsheng Ou, Xinping Wu, Gaolei Jia, Xiaoqin Qian

**Affiliations:** ^1^ Department of Ultrasound, The Affiliated People’s Hospital of Jiangsu University, Zhenjiang, Jiangsu, China; ^2^ Department of Ultrasound, Traditional Chinese Medicine Hospital of Nanjing Lishui District, Nanjing, Jiangsu, China; ^3^ Department of Thyroid Surgery, The Affiliated People’s Hospital of Jiangsu University, Zhenjiang, Jiangsu, China; ^4^ Department of Geriatrics, The Affiliated People’s Hospital of Jiangsu University, Zhenjiang, Jiangsu, China; ^5^ Laboratory Center, The Affiliated People’s Hospital of Jiangsu University, Zhenjiang, Jiangsu, China; ^6^ Department of Ophthalmology, The Affiliated People’s Hospital of Jiangsu University, Zhenjiang, Jiangsu, China; ^7^ Department of Endocrinology, Yancheng city No.6 People’s Hospital, Yancheng, Jiangsu, China; ^8^ Department of Pathology, The Affiliated People’s Hospital of Jiangsu University, Zhenjiang, Jiangsu, China; ^9^ Department of Cardiology, The Affiliated People’s Hospital of Jiangsu University, Zhenjiang, Jiangsu, China; ^10^ Medical Marketing Department, Nanjing D.A. Medical Laboratory, Nanjing, Jiangsu, China; ^11^ Department of Ultrasound, Jiangsu Hospital of Integrated Traditional Chinese and Western Medicine, Nanjing, Jiangsu, China; ^12^ Department of Vascular Thyroid Hernia Surgery, Xuzhou Central Hospital, Xuzhou Clinical School of Xuzhou Medical University, Xuzhou, Jiangsu, China; ^13^ Department of Ultrasound Medicine, Northern Jiangsu People’s Hospital Affiliated to Yangzhou University, Yangzhou, Jiangsu, China

**Keywords:** thyroid cancer, children and adolescents, adults, genomic alterations, clinical ultrasound manifestations

## Abstract

**Introduction:**

Papillary thyroid carcinoma is the most common pathological subtype of thyroid cancer in both children/adolescents (TCCA) and adults (TCA). TCCA manifests more aggressive and invasive behaviors than TCA, which may be attributed to specific genomic alterations.

**Methods:**

To better understand the specific molecular, pathological and clinical manifestations of TCCA, we retrospectively analyzed a cohort of 60 patients with sporadic papillary thyroid carcinoma, including 20 TCCAs and 40 TCAs. Fine-needle aspiration tissue samples from these cases were analyzed using next-generation sequencing. Demographics, ultrasound features, postoperative pathology and radiation exposure history were compared between TCCAs and TCAs. To validate our findings, we integrated data from 28 prior studies, resulting in a larger cohort of 1,483 sporadic TCCAs.

**Results:**

Multiple gene mutations were more prevalent in TCCAs than TCAs (p=0.013), such as *BRAF^V600E^* coexisting with KMT2 family genes or PTEN. Although *BRAF^V600E^* was the most common single nucleotide variant in TCCAs (25%, 5/20), its prevalence was significantly lower than in TCAs (95%, 38/40, p<0.0001). *RET* oncogenic fusions were detected exclusively in TCCAs, with an incidence of 20% (4/20). Compared with TCAs, TCCAs were associated with larger tumor diameters (p<0.001), more advanced tumor staging (T3–T4, p<0.001; N2, p=0.002), higher incidence of extrathyroidal extension (TCCA: 25%, TCA: 5%, p=0.036) and more frequent lymph node metastasis (TCCA: 70%, TCA: 27.5%, p=0.0024). Importantly, TCCAs harboring *BRAF^V600E^* alongside other mutations (e.g., *ATM, PTEN* or *KMT2* family genes) exhibited more severe clinical manifestations, including larger tumors and higher rates of lymph node metastasis, compared with those harboring *BRAF^V600E^* alone.

**Discussion:**

TCCAs exhibit more aggressive and invasive clinical manifestations than TCAs, particularly in cases with *RET* fusions or *BRAF^V600E^* coexisting with other point mutations. Targeted comprehensive molecular profiling may aid in the diagnosis and treatment of TCCA.

## 1 Introduction

Thyroid cancer is the most common endocrine malignancy, with rising incidence in both pediatric and adult populations (1). It is the second most common malignant neoplasm in adolescents worldwide, with an annual incidence of 0.44/100,000 and a mortality rate of 0.02/100,000 in children and adolescents in China (2–5). To account for differences in clinicopathological characteristics and management strategies, the American Thyroid Association classifies thyroid cancer into thyroid cancer in children and adolescents (TCCA, age ≤18) and in adults (TCA, age >18) (6).

Although TCCA management is largely based on TCA guidelines due to shared pathological features (7–9), TCCAs exhibit distinct clinical behaviors, pathophysiology, and long-term outcomes (10). TCCAs are generally more aggressive, often presenting with multifocality, extrathyroidal invasion, and direct involvement of the recurrent laryngeal nerve, trachea, blood vessels and esophagus (11). Moreover, while thyroid nodules are more common in TCAs (19–68%), only 5–10% are malignant. In contrast, nodules are less common in TCCAs (1–3%) but carry a significantly higher malignancy rate (22–26%), highlighting the need for pediatric-specific diagnostic and therapeutic strategies.

With the rapid advancement of next-generation sequencing (NGS) (12), many studies have characterized the genomic landscape of TCA (13–15). Common genomic alterations, such as *BRAF^V600E^*, have been identified and widely used to diagnose TCA (16, 17). However, current guidelines are primarily based on adult data and may not capture the unique genomic features of TCCA. The genetic underpinnings of TCCA remain underexplored, despite their relevance to clinical behavior and treatment. Therefore, investigating these differences is essential to improve diagnostic accuracy and inform tailored therapeutic approaches.

In this study, we compared the genomic alterations between TCCAs and TCAs in an in-house cohort and analyzed the differences in their clinicopathological and ultrasound characteristics. The findings were validated in a large cohort of 1,483 sporadic TCCAs from 28 published studies. Our findings underscored the importance of genomic detection in predicting outcomes and informing therapeutic guidelines for TCCA.

## 2 Materials and methods

### 2.1 Ethics declarations

The study was conducted in accordance with the Declaration of Helsinki and was approved by the Ethics Committee of Affiliated People’s Hospital of Jiangsu University, Traditional Chinese Medicine Hospital of Nanjing Lishui District (IRB approval number: 2022LW037), and Xuzhou Central Hospital (XZXY-LK-20230314-027), Xuzhou Clinical School of Xuzhou Medical University (K-20230090-W). All samples were collected with written informed consent and relevant institutional approval.

### 2.2 Patient selection

**Figure 1:**
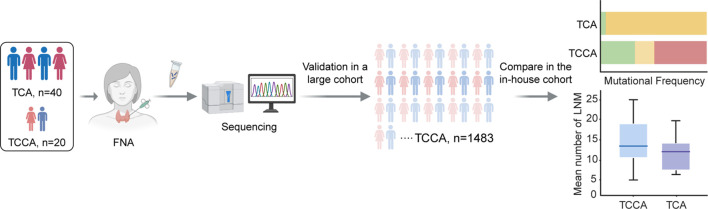
Schematic illustration of workflow in this study. FNA, fine-needle aspiration; TCA, thyroid cancer in adults; TCCA, thyroid cancer in children and adolescents.

Twenty TCCAs and forty TCAs with a histopathological diagnosis of papillary thyroid cancer (PTC) were recruited from the Traditional Chinese Medicine Hospital of Nanjing Lishui District, Xuzhou Central Hospital and Affiliated People’s Hospital of Jiangsu University between 2021 and 2023. Retrospectively collected data included demographics (age, sex, and radiation exposure history), NGS-detected genomic alterations, histopathologic subtypes, maximum tumor diameters, extrathyroidal extension (ETE), multifocality, lymph node metastases (LNM), distant metastases (DM), and treatment history. A schematic illustration of the study workflow is presented in **Figure 1**.

Minimal extrathyroidal extension (ETE, T3) refers to microscopic invasion into the perithyroidal soft tissues or sternothyroid muscle, whereas extensive ETE (T4a) involves macroscopic invasion into structures such as the subcutaneous tissues, larynx, trachea, esophagus, or recurrent laryngeal nerve. Extensive ETE is typically identified via preoperative ultrasound, CT, or MRI, and confirmed by pathological examination after surgery. LNM is suspected when ultrasound reveals features such as microcalcifications, cystic changes, peripheral vascularity, hyperechogenicity, or a round shape. Definitive diagnosis requires ultrasound-guided fine-needle aspiration cytology of the washout fluid, with biopsy recommended for central nodes ≥8 mm and lateral nodes ≥10 mm. Chest CT, whole-body radioactive iodine scans, and F-fluorodeoxyglucose positron emission tomography/CT are used to detect distant metastases, particularly in the lungs, bones, and liver. Pathological confirmation through surgical resection or biopsy remains essential, as ETE in lymph nodes and vascular invasion in the primary tumor influence risk stratification. To enhance diagnostic precision and inform staging, patient-specific factors such as tumor size, multifocality, and molecular markers (e.g., *BRAF* mutations) were also considered.

### 2.3 Selection criteria for the large TCCA cohort from prior studies

We identified 108 relevant studies, with 104 from PubMed and 4 from Cochrane Library database. After removing duplicates and excluding 57 studies due to non-TCCA genomic alterations, 28 studies were included (**Supplementary Figure 1**; **Supplementary Table 1**). To ensure that all TCCAs were sporadic cases, 31 cases with radiation exposure history were excluded. Ultimately, a large cohort of 1,483 TCCAs was included in the analysis.

### 2.4 Ultrasound imaging

Experienced ultrasound radiologists performed preoperative standard ultrasound scans on the patients using a GE LOGIQ s8, LOGIQ E9, LOGIQ E20 (American General’s GE Medical Systems), Mindray Resona 7, Philips Q5 (Healthcare, Eindhoven, the Netherlands), Philips iU22 ultrasound machine equipped with a 5–12 MHz linear array transducer. These machines were operated using a program specifically designed for thyroid imaging.

During the procedure, patients were positioned horizontally without a pillow, with their heads slightly inclined to maximize the exposure of the cervical region. Ultrasound assessments of the thyroid and surrounding neck structures were conducted using both cross-sectional and longitudinal scanning. Key ultrasound characteristics assessed included tumor diameter (maximum long axis of the lesion), multifocality (singleness or multifocality), aspect ratio (height divided by width on transverse views, A/T), tumor shape (irregular or regular), tumor border (clear or fuzzy), internal echo pattern (uniform, nonuniform), tumor vascularization (none, rare or abundant), and ultrasonic diagnosis of ETE (positive or negative) and LNM (positive or negative).

### 2.5 Molecular genetic analysis

#### 2.5.1 Total nucleic acid extraction

Total DNA and RNA were extracted from fine-needle aspiration biopsy (FNAB) samples using the QIAamp DNA Mini Kit (QIAGEN, Germany) following the manufacturer’s protocol, and quantified using Nanodrop 1000 (Thermos Fisher, USA).

#### 2.5.2 Library construction

For quality control, at least 10 ng of total DNA of each sample was extracted and fragmented. Q30 rate of DNA base was above 80%, and DNA sequence alignment rate was above 90%. Mean sequencing depth was over 500×. Q30 rate of RNA base was above 80%, and total reads in the RNA target region was 300,000 reads.

Genomic DNA sequencing libraries were created using the TruSeq DNA Library Preparation Kit following the manufacturer’s protocol. DNA sequencing was performed on the Illumina NextSeq sequencing system (San Diego, CA), using 2100 bp peer read. BWA (Burrows-Wheeler aligner) 10 was used to compare reads with the human genome construction GRCh37. MuTect2 (3.4-46-gbc02625) 11 was used to identify single nucleotide variants (SNVs) and GATK was used to identify small insertions and deletions. Final candidate variants were confirmed with the Integrated Genomics Viewer browser.

#### 2.5.3 NGS

Genetic variations of FNAB samples were detected by NGS (DIAN Diagnostics), including SNVs, fusion alterations (FAs) and copy number alterations. Sixty samples per NGS Illumina NextSeq run were pooled in an equimolar ratio 6 pM library pool. Targeted deep sequencing including a 6% PhiX control library was prepared for sequencing according to the Illumina NextSeq System user guide (Illumina, San Diego, CA). Subsequently, sequencing was carried out on a NextSeq instrument (Illumina) using the v2 chemistry as recommended by the manufacturer.

#### 2.5.4 Statistical analysis

All statistical analyses were carried out using IBM SPSS statistical software (statistic 26.0 version; IBM SPSS, Armonk, NY, USA) or R-4.2.0. The continuous variables between two groups were compared using the Wilcoxon rank-sum test, and Fisher’s exact test was used to compare the categorical variables. All statistical tests were two-sided, and p<0.05 was considered statistically significant.

## 3 Results

### 3.1 TCCAs exhibit more aggressive and invasive clinical features than TCAs

**Figure 2:**
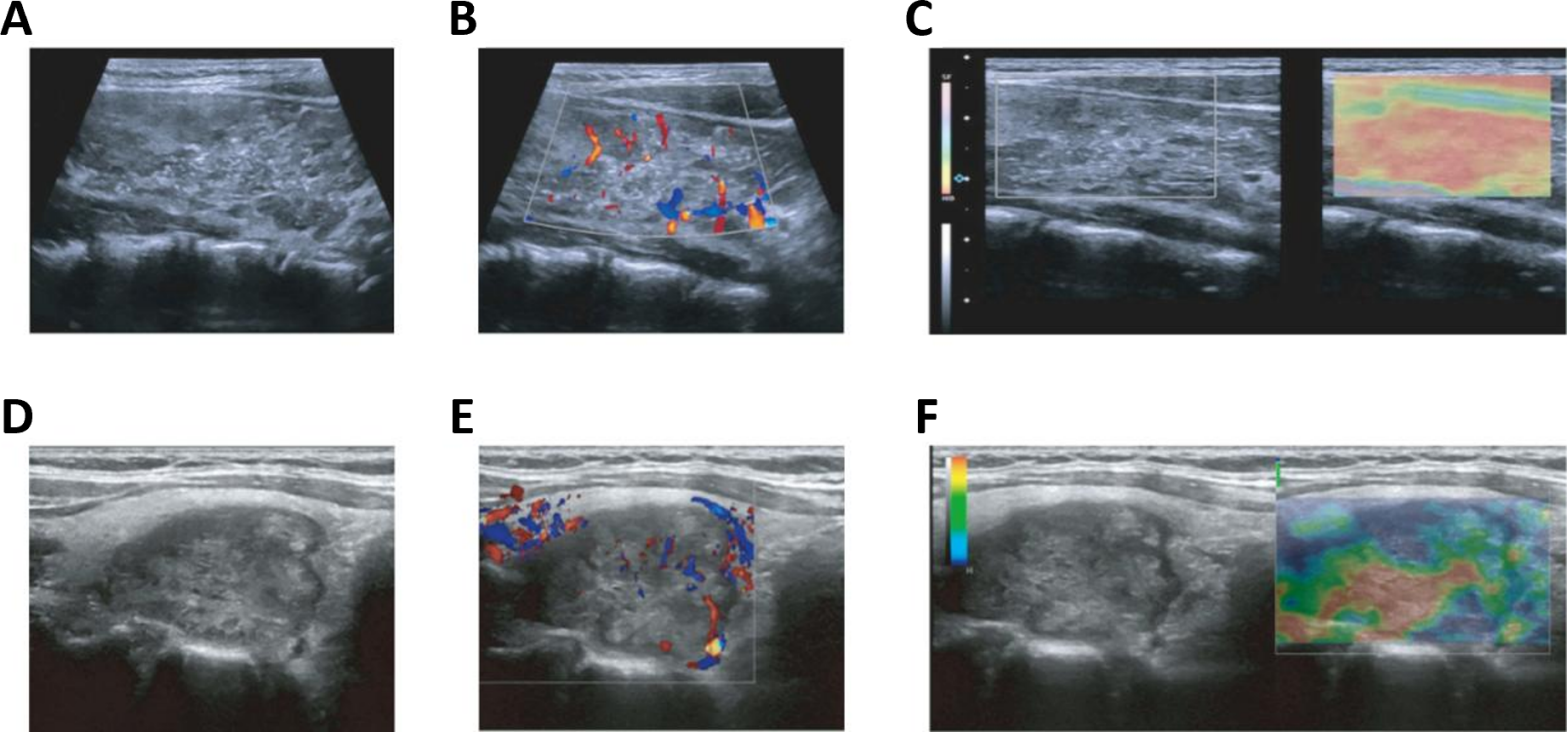
Ultrasound images of TCCAs and TCAs. **(A–F)** Multimodal ultrasound images of a 15-year-old female **(A–C)** and a 44-year-old male **(D–F)** with PTC. Postoperative pathology confirmed TCCA in the female patient and TCA in the male patient. PTC, papillary thyroid cancer; TCA, thyroid cancer in adults; TCCA, thyroid cancer in children and adolescents. Statistical significance: *p<0.05, **p<0.01, ***p<0.001.

Given the significance of ultrasonography in thyroid carcinoma detection, we first investigated differences in ultrasound imaging characteristics between TCCAs and TCAs. TCCAs had larger tumor diameters than TCAs (p<0.001), with no significant differences in other ultrasound characteristics (**Tables 1**, **2**). Also, lesions in TCCAs appeared relatively hyperechoic, with diffusely distributed microcalcifications, irregular morphology, and fuzzy borders on the grayscale ultrasound image (**Figures 2A–C**). In contrast, TCAs were hypoechoic with irregular morphology (**Figures 2A, D**). Color Doppler imaging showed more abundant vascularization inside the nodules in TCCAs than in TCAs (**Figures 2B, E**). Elastography images confirmed the nodules were harder than TCAs, with elastic scores of ≥3 (**Figures 2C, F**).

**Table 1:** Comparison of ultrasound imaging characteristics between TCCAs and TCAs.

Variable	TCCAs (n = 20)	TCAs (n = 40)	P-value
Aspect Radio-no. (%)			0.088
<1	16 (80)	21 (52)	
>1	4 (20)	14 (35)	
=1	0 (0)	5 (12)	
Tumor Shape-no. (%)			0.27
Irregular	11 (55)	15 (38)	
Regular	9 (45)	25 (62)	
Tumor Border-no (%)			0.17
Clear	11 (55)	14 (35)	
Fuzzy	9 (45)	26 (65)	
Internal Echo Pattern-no (%)			0.33
Nonuniform	19 (95)	40 (100)	
Uniform	1 (5)	0 (0)	
Tumor Vascularization-no (%)			0.78
Rare	12 (60)	26 (65)	
Abundant	8 (40)	14 (35)	

TCA, thyroid cancer in adults; TCCA, thyroid cancer in children and adolescents.

**Table 2:** Comparison of clinical manifestations between TCCAs and TCAs.

Characteristics	TCCAs (n=20)	TCAs (n=40)	P-value
Age (year) - median (IQR)	16 (13.8–17.0)	47 (34.8–57.3)	**<0.001**
Sex - no. (%)			
Female	13 (65)	30 (75)	0.540
Male	7 (35)	10 (25)	
Tumor diameter (mm)	11.4	23.5	**<0.001**
Size - no. (%)			
>20 mm	13 (65)	3 (7.5)	**<0.001**
≤20 mm	7 (35)	37 (92.5)	
Prognostic stages (%)			
I	20 (100)	40 (100)	NS
II	0 (0)	0 (0)	
III	0 (0)	0 (0)	
IV	0 (0)	0 (0)	
TNM stages (%)			
T			
T1–T2	10 (50)	40 (100)	**<0.001**
T3–T4	10 (50)	0 (0)	
N			
Nx	5 (25)	28 (70)	**0.002**
N1	1 (5)	1 (2.5)	
N2	14 (70)	11 (28)	
M			
M0	20 (100)	40 (100)	NS
M1	0 (0)	0 (0)	
Multifocality - no. (%)			
Multifocality	12 (60)	16 (40)	0.176
Singleness	8 (40)	24 (60)	
ETE - no. (%)			
Positive	5 (25)	2 (5)	**0.036**
Negative	15 (75)	38 (95)	
LNM - no. (%)			
Positive	15 (75)	11 (28)	**0.002**
Negative	5 (25)	29 (72)	
Mean number of LNM	1.4	7.7	**<0.001**

ETE, extrathyroidal extension; IQR, interquartile range; LNM, lymph node metastases; TCA, thyroid cancer in adults; TCCA, thyroid cancer in children and adolescents; TNM, tumor node metastasis. Bold values denote statistical significance (p<0.05).

**Figure 3:**
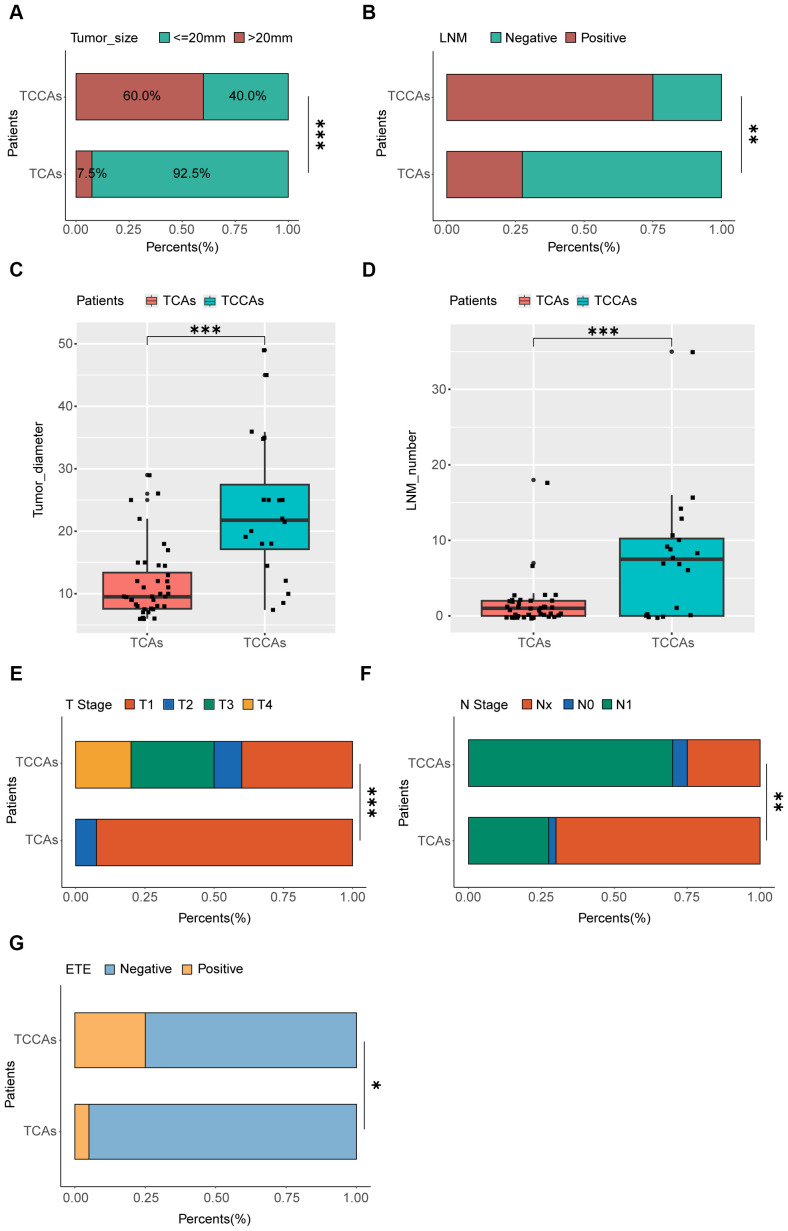
Comparison of clinic-pathological manifestations between TCCAs and TCAs. **(A)** Proportions of patients with different tumor sizes among the TCCAs and TCAs. **(B)** Proportions of patients with LNM among the TCCAs and TCAs. **(C)** Boxplot showing the tumor diameters among TCCAs and TCAs. Wilcoxon rank-sum test was used to measure the differences between groups. **(D)** Boxplot showing the number of positive lymph nodes among TCCAs and TCAs. Wilcoxon rank-sum test was used to measure the differences between groups. **(E)** Proportions of patients with T stage among the TCCAs and TCAs. **(F)** Proportions of patients with N stage among the TCCAs and TCAs. **(G)** Proportions of patients with ETE among the TCCAs and TCAs. ETE, extrathyroidal extension; LNM, lymph node metastases; TCAs, thyroid cancer in adults; TCCAs, thyroid cancer in children and adolescents.

Analysis of baseline characteristics and clinical manifestations showed no significant differences between TCCAs and TCAs in sex (p=0.54) and multifocality (p=0.176) (**Table 2**). However, TCCAs exhibited more aggressive and invasive clinical features, including larger tumor sizes (p<0.001), more advanced tumor staging (T3–T4, p<0.001; N2, p=0.002), higher incidence of ETE (TCCA: 25%, TCA: 5%, p=0.036), LNM (TCCA: 75%, TCA: 28%, p=0.002) and more positive lymph nodes (p<0.001) (**Figure 3**). To validate these findings, we analyzed clinical manifestations in the large cohort. As expected, consistent with TCAs, classical PTC was the dominant histopathologic subtype of TCCAs (667/1483, 45%), followed by the follicular variant of PTC (291/1483, 19.6%) (**Table 3**). Nearly half of TCCAs exhibited LNM (745/1483, 50.2%) or DM (151/1483, 10.2%), 22.7% harbored ETE, and 19.9% had multifocality, indicating the aggressiveness and invasiveness of TCCAs (**Table 3**).

**Table 3:** Clinical data in previous studies of TCCAs.

Parameters	Female	Ethnicity		Pathology								ETE	Multifocality	LNM	DM
		Caucasian	East Asian	cPTC	fvPTC	dsvPTC	svPTC	hsvPTC	Other PTC subtypes	FTC	MTC, WDTC, ATC				
Total patients (n=1483 cases)	1048 (70.7%)	1141 (76.9%)	342 (23.1%)	667 (45.0%)	291 (19.6%)	63 (4.2%)	37 (2.5%)	3 (0.2%)	102 (6.9%)	45 (3.0%)	5 (0.3%)	337 (22.7%)	295 (19.9%)	745 (50.2%)	151 (10.2%)

Articles are listed in the **Supplementary Table 1**.

ATC, anaplastic thyroid cancer; cPTC, classical papillary thyroid carcinoma; DM, distant metastases; dsvPTC, diffuse sclerosing variant of papillary thyroid carcinoma; ETE, extrathyroidal extension; FTC, follicular thyroid carcinoma; fvPTC, follicular variant papillary thyroid carcinoma; hsvPTC, hobnail variant of papillary thyroid carcinoma; LNM, lymph node metastases; MTC, medullary thyroid cancer; PTC, papillary thyroid carcinoma; svPTC, solid variant papillary thyroid carcinoma; TCCA, thyroid cancer in children and adolescents; WDTC, well-differentiated thyroid carcinoma.

### 3.2 TCCAs harbor distinct genomic alterations compared to TCAs

**Figure 4:**
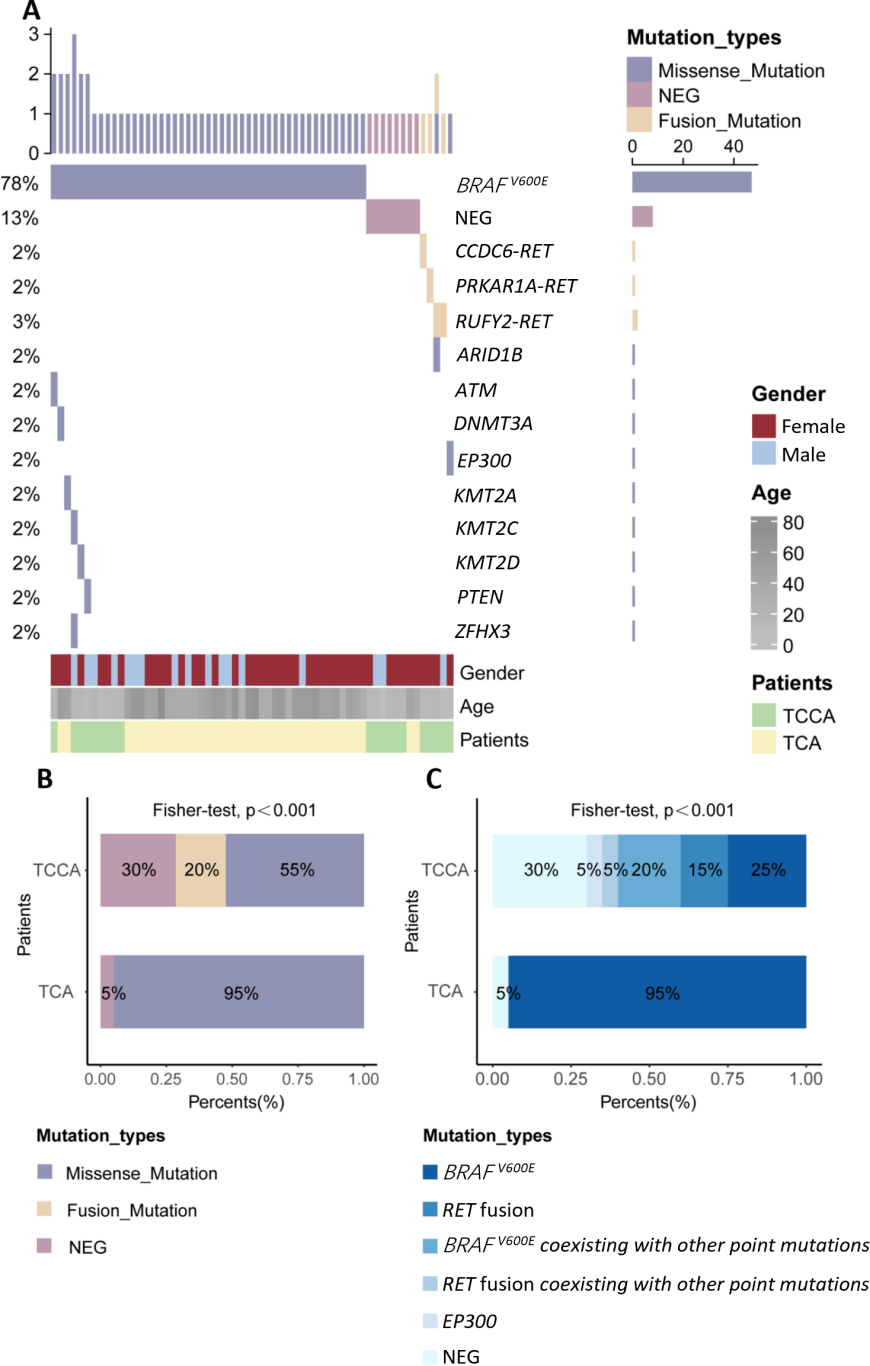
Genomic landscape of TCCAs and TCAs in this in-house cohort. **(A)** Waterfall Plots showing gene mutations of TCCAs and TCAs in this in-house cohort; **(B)** Bar plot showing the frequencies of detected mutation types in TCCAs and TCAs; **(C)** Proportions of detected gene mutations in TCCAs and TCAs in this in-house cohort. NEG, negative; TCAs, thyroid cancer in adults; TCCAs, thyroid cancer in children and adolescents.

We compared mutation types and frequencies between TCCAs and TCAs in our in-house cohort and validated these findings in the large cohort (**Supplementary Figure 1**, **Supplementary Table 1**). The genomic alterations and clinical characteristics of 40 TCAs (30 females and 10 males) and 20 TCCAs (13 girls and 7 boys) are presented in **Figure 4**; **Supplementary Table 2**. The distribution of genomic alterations in the large cohort is presented in **Table 4**.

**Table 4:** Genetic variations in TCCAs in the 28 previous studies.

Genetic variations	Single nucleotide variants				Fusion alterations		
	*BRAF*	*RAS*	*DICER1*	Others	*RET*	*NTRK*	Others
Total cases with genetic variations (n=850 cases)	396 (46.6%)	71 (8.4%)	18 (2.1%)	19 (2.2%)	227 (26.7%)	78 (9.2%)	41 (4.8%)

Articles are listed in the **Supplementary Table 1**.

TCCA, thyroid cancer in children and adolescents.

Approximately 70% (14/20) of TCCAs in our in-house cohort harbored genetic alterations, higher than the 57.3% (850/1,483) in the large cohort. *BRAF^V600E^* was the most common SNV in TCCAs, found in 46.6% (396/850) of the cases in the large cohort, followed by *RET* oncogenic fusions (26.7%, 227/850) (**Table 4**). Consistently, in the in-house cohort, 45% (9/20) of TCCAs had *BRAF^V600E^* (five with *BRAF^V600E^* alone and four with *BRAF^V600E^* and other mutations), and 20% (4/20) had *RET* fusion. *RAS* (8.4%, 71/850), *DICER1* (2.1%, 18/850) and *NTRK* (9.2%, 78/850) alterations, reported in the large TCCA cohort, were absent in our in-house cohort (**Tables 4**; **Table 5**). Notably, N-methyltransferase 2 (*KMT2*) gene family mutations (e.g., *KMT2C* and *KMT2D*) coexisting with *BRAF^V600E^* were detected only in TCCAs in our in-house cohort. One case each of *PTEN* and *EP300* mutation was found in our in-house cohort (**Figure 4A**).

**Table 5:** Comparison of genetic alternations between TCCAs and TCAs.

Mutations	TCCAs (n=20)	TCAs (n=40)	Total (n=60)	P-value
*BRAF^V600E^*	5 (25%)	36 (90%)	41 (68.3%)	<0.0001
*BRAF^V600E^* coexisting with other point mutations	4 (20%)	2 (5%)	6 (10%)	0.089
*RAS*	0	0	0	>0.99
*DICER1*	0	0	0	>0.99
*RET* fusion	4 (20%)	0	4 (6.7%)	0.01
*NTRK* fusion	0	0	0	>0.99
Other gene mutations	1 (5%)	0	1 (1.7%)	0.33
Negative	6 (30%)	2 (5%)	8 (13.3%)	0.013
Total (cases)	20	40	60	

TCA, thyroid cancer in adults; TCCA, thyroid cancer in children and adolescents.

Furthermore, we compared genetic variants between TCCAs and TCAs (**Table 5**, **Figures 4B, C**). *BRAF^V600E^* was significantly more prevalent in TCAs (36/40, 90.0%) than in TCCAs (9/20, 45%, p<0.0001). Notably, *RET* oncogenic fusions were detected in 20% (4/20) of TCCAs but absent in TCAs (p=0.01), highlighting *RET* fusions as TCCA-specific events. Additionally, uncommon gene mutations included one case of *KMT2A* in TCA and one case each of *KMT2C* and *KMT2D* in TCCA.

### 3.3 TCCAs with *BRAF^V600E^* and other point mutations exhibited more severe clinical manifestations than those with *BRAF^V600E^* alone

Although the mutational frequency of *BRAF^V600E^* alone was comparable to that of *BRAF^V600E^* coexisting with other point mutations, TCCAs harboring *BRAF^V600E^* and other point mutations manifested larger tumor diameters and size (p=0.048) than those with *BRAF^V600E^* alone. Moreover, TCCAs with multiple gene mutations had more LNMs, as reflected by more positive LNMs (p=0.048) and a greater mean number of LNMs (p=0.017). No significant differences were observed in multifocality or ETE (**Table 6**).

**Table 6:** Comparison of **clinical manifestations between TCCAs with *BRAF^V600E^*** and *BRAF^V600E^* coexisting with other gene mutations.

Characteristics	*BRAF^V600E^*	*BRAF^V600E^* coexisting with other point mutations	P-value
Tumor diameter (mm)	16.1	32.0	**0.048**
Size - no. (%)			
>20 mm	1 (20)	4 (100)	**0.048**
≤20 mm	4 (80)	0 (0)	
Multifocality - no. (%)			
Multifocality	1 (20)	3 (75)	1.000
Singleness	4 (80)	1 (25)	
ETE - no. (%)			
Positive	1 (20)	1 (25)	1.000
Negative	4 (80)	3 (75)	
LNM - no. (%)			
Positive	4 (80)	4 (100)	**0.048**
Negative	1 (20)	0 (0)	
Mean number of LNM	7 (0–8.0)	12.5 (10.8–14.5)	**0.017**

ETE, extrathyroidal extension; LNM, lymph node metastases; TCCA, thyroid cancer in children and adolescents. Bold values denote statistical significance (p<0.05).

## 4 Discussion

### 4.1 TCCAs manifested more aggressive and invasive clinical features than TCAs, particularly in cases with *RET* fusions or *BRAF^V600E^* co-mutations

A 2021 population-based study reported incidence rates of TCCA ranged from 0.4–13.4 cases per 1 million person-years (2), primarily due to the diverse PTC subtypes. Incidence increases with age and is higher in girls than in boys. Similarly, in our in-house cohort, female cases outnumbered male cases in both TCCA and TCA.

TCCAs exhibit distinct ultrasound features compared to TCAs. While both groups commonly showed microcalcifications and irregular margins, TCCAs were more likely to present with lymph node involvement, whereas an aspect ratio >1 was more typical of TCAs (18). Hypoechoic features are not specific in the diagnosis of TCA and TCCA. In our study, TCCAs had significantly larger tumors than TCAs (p<0.001), although other ultrasound features did not differ significantly. TCCAs also demonstrated more aggressive histopathological features, including higher rates of ETE, LNM, and multifocality. These nodules often appeared hyperechoic with irregular morphology, diffuse microcalcifications, and increased vascularity. PTC is typically multifocal, aggressive, with a strong tendency to invade the thyroid capsule and directly involve surrounding structures such as the laryngeal nerve, trachea, blood vessels and esophagus (4, 19). Microcalcifications and suspicious cervical lymph nodes are highly indicative of PTC, whereas benign lesions are more likely to be cystic, hyperechoic, and have regular margins with marginal blood flow. Suspicious lymph nodes are typically evaluated based on size, shape, disappearance of lymphatic portal, strong echo, cystic changes, microcalcification and increased blood flow. While microcalcification and increased blood flow have the highest specificity, their sensitivity remains low (11). Notably, no single ultrasound feature is sufficient to assess the malignancy of a thyroid nodule or determine LNM. To improve diagnostic accuracy, we performed ultrasound-guided FNAB, the current preferred preoperative diagnostic tool, especially in children with nodules >1 cm, normal or low thyroid function, and suspicious ultrasonographic features (10, 18). While nodule size may be influenced by age-related changes, our findings suggest that TCCAs exhibit more aggressive clinical features than TCAs.

The malignancy rates of thyroid nodules are significantly higher in TCCAs (22–26%) than in TCA (5–10%) (20, 21). In our study, nearly half of the TCCAs in the large cohort had LNM, 9.7% had DM, 21.7% had ETE, and 19.0% had multifocality, consistent with the in-house cohort. These rates are consistent with previous studies reporting higher recurrence risk (40–80%) and metastatic potential in TCCAs compared to TCAs (18).

TCCAs exhibit more aggressive and invasive clinical features than TCAs, especially when *BRAF^V600E^* coexists with other mutations, primarily due to synergistic activation of oncogenic pathways. In children, the developing thyroid microenvironment is more susceptible to epigenetic reprogramming by co-mutations, enhancing proliferation, apoptosis, evasion, and angiogenesis. For instance, *BRAF^V600E^* activates the MAPK pathway, while *TERT* promoter mutations increase telomerase activity, accelerating tumor growth and invasion. Additionally, higher metabolic and growth factor signaling in children may further amplify these effects, promoting rapid tumor expansion and lymph node metastasis. These interactions are less frequent in adults, contributing to clinical differences by age.

Multi-gene panel testing helps identify high-risk cases, as co-mutations often correlate with classic PTC histology and extrathyroidal extension, guiding aggressive staging. Treatment may require total thyroidectomy, higher-dose radioactive iodine, or adjuvant targeted therapies (e.g., BRAF/MEK inhibitors plus TERT pathway modulators) in resistant or advanced cases. Co-mutated tumors have higher recurrence and metastasis risk, necessitating close long-term surveillance with imaging and molecular monitoring. Understanding these mutational profiles allows for personalized risk stratification, ensuring pediatric patients receive targeted treatments that improve survival and reduce treatment-related complications.

Overall, these findings suggest that TCCAs exhibit more aggressive and invasive clinicopathological features than TCAs, highlighting the importance of early identification.

### 4.2 The genomic landscape of TCCAs requires comprehensive consideration beyond *RET* and *NTRK* fusions

Genetic alterations play a critical role in the clinicopathological features of TCCAs, highlighting the need for a comprehensive understanding of its genomic landscape to inform specific management guidelines. However, previous studies on TCCAs often focus solely on FAs or copy number alterations due to childhood radiation exposure from events like Chernobyl and Fukushima accidents (22–24). Additionally, earlier studies primarily focused on the common SNVs (e.g., *BRAF^V600E^*) or FAs (e.g., *RET*) (25, 26), overlooking other critical tumor-suppressor gene mutations. Recent large-cohort studies in thyroid cancer have highlighted additional genetic aberrations relevant to immunotherapy (27, 28). In our analysis of 1,483 TCCAs from 28 prior studies, *BRAF^V600E^*, previously considered less frequent in TCCAs (29), was present in 46.6% (396/850) of cases. *RAS* mutations appeared mainly in the follicular variant PTC and follicular thyroid carcinoma, while *RET* and *NTRK* fusions remained common FAs, consistent with previous literature.

In our in-house cohort, the detection rate of genomic alterations was 57.3% (70.0% for TCCAs and 95.0% for TCAs). Although *BRAF^V600E^* is the dominant single point mutation in TCA with PTC cases, its prevalence and link to aggressive features in sporadic TCCAs remain understudied (25, 29–32). A study of 19 classical PTC TCCAs reported a 63.6% *BRAF^V600E^* prevalence, higher than the 45% and 50.9% reported in two meta-analyses of TCAs (19). Consistently, our study reported a higher incidence of *BRAF^V600E^* in TCAs (90.0%, 36/40) than that in TCCAs (19). Also, *BRAF^V600E^* was the most prevalent genomic alteration in TCCAs (45%, 9/20), lower than the large cohort (46.6%) and TCAs (90%). TCCAs with *BRAF^V600E^* are usually at a more advanced stage. Only two U.S. studies reported the prevalence of *BRAF^V600E^* in TCCAs, without reviewing aggressive clinical characteristics. Geographic variation may influence oncogene mutation rates (33, 34), and our study contributes valuable data on the genetic mutation landscape of TCCAs in China.

*RAS*, *BRAF, RET/PTC* and *PAX8/PPARg* mutations are strongly associated with TCCA malignancy, especially in cases with uncertain FNAB cytology (35). The presence of these mutations can increase the positive predictive value of FNAB, with overall sensitivity and specificity reaching 80% and 100%. Notably, *RET* oncogenic fusions were detected exclusively in TCCAs (4/20, 20%), compared to a much lower prevalence (1.69%) in a large TCA cohort, suggesting these fusions are TCCA-specific (36). *RET* fusions are primarily associated with sporadic and radiation-induced PTCs (37–40). In younger patients, *NCOA4-RET* fusion is more prevalent in early radiation-related cases, while *CCDC6-RET* is more prevalent in sporadic cases (41). In this study, all TCCAs harboring *RET* fusions (one *CCDC6-RET*, two *RUFY2-RET* and one *PRKAR1A-RET*) were sporadic, consistent with previous studies (36).

The carcinogenic mechanism of *RET* fusion involves ligand-independent dimerization and constitutive activation of RET kinase, which drives tumorigenesis through key signaling pathways, including MAPK, PI3K, JAX-STAT, PKA, and PKC signaling pathways, resulting in excessive cell proliferation (42, 43). In addition, kinase activation caused by *RET* fusion promotes cytokine production, contributing to specific biological phenotypes and facilitating tumor initiation and progression (37, 44). Therefore, TCCAs with RET fusions, particularly *CCDC6-RET*, *NCOA4-RET*, and *KIF5B-RET*, exhibit more aggressive and invasive clinicopathological features due to this enhanced carcinogenic activity (45–47).

Notably, although less frequent, *KMT2* and *PTEN* coexisting with *BRAF^V600E^* were detected in TCCAs in our in-house cohort and should not be overlooked. The KMT2 family mainly includes KMT2A, KMT2B, KMT2C and KMT2D. KMT2A and KMT2B regulate gene expression by depositing H3K4me2 and H3K4me3 marks at gene promoters, upregulating target genes and potentially promoting tumorigenesis through various signaling pathways. Overexpression of *KMT2A* has been implicated in thyroid cancer, with its knockdown suppressing thyroid cancer cell proliferation. The National Cancer Institute Genomic Data Commons analyzed more than 33,000 cases and identified *KMT2D* and *KMT2C* as the third and seventh most frequently mutated cancer genes. Most mutations are nonsense or frameshift, causing early translation termination and reduced protein expression. While further research is needed to clarify the role of KMT2 mutations in TCCA, these mutations warrant attention.

### 4.3 Tumor mutational burden from multiple gene mutations may drive aggressive clinical features

In this in-house cohort, TCCAs with *BRAF^V600E^* combined with additional mutations such as *ATM*, *PTEN* or *KMT2* family genes exhibited more aggressive clinical features, including larger tumor size and higher rates of LNM. *BRAF^V600E^* coexisting with other point mutations such as *PTEN*, *EP300* or *TP53* has been linked to poorer prognosis in PTC (48). Our prior study also demonstrated that *BRAF^V600E^* coexisting with *TERT* promoter mutations contributes to adverse clinical outcomes in radioiodine-refractory differentiated thyroid cancer (49). *BRAF^V600E^* with additional mutations is associated with increased tumor mutational burden, which may promote recurrence by altering the immune microenvironment, reducing CD8+ T cells and M1 macrophages (50). These findings underscore that focusing solely on common gene variants such as *RET* or *NTRK* in the early stage is insufficient for predicting clinical outcomes in TCCAs. Comprehensive molecular profiling is essential for accurate prognosis and treatment planning.

In summary, TCCAs exhibit distinct genomic alterations, particularly *RET* fusions, compared with TCAs, consistent with previous reports. In both our in-house and large cohort, *BRAF^V600E^* was more frequently observed in TCCAs than in previous reports (29), although it was lower than TCAs in our cohort. Importantly, tumor mutational burden from multiple gene mutations, such as *BRAF^V600E^* coexisting with *KMT2* or *PTEN*, should be considered in TCCAs. Overall, in contrast to previous reports (32, 37), our findings underscore the need for a comprehensive analysis of genomic alterations in TCCAs, beyond *RET* and *NTRK*.

This study has several limitations. First, the small number of pediatric tissue samples may impact the robustness of the findings, necessitating validation in a larger cohort. Second, despite the broad coverage of our NGS panel, undetected driver alterations in the six NGS-negative samples may have led to an underestimation of the overall detection rate. Third, the NGS method used was unable to detect structural variants, leaving the potential role of these alterations unexplored. Future studies should consider using complementary approaches to capture structural variants and provide a more complete picture of the genetic landscape in thyroid cancer. Nonetheless, to address this limitation, we validated our findings in a large external cohort of 1,483 TCCAs and compared them with TCAs. Our study thus offers valuable insights into the specific genomic alterations and aggressive features of TCCAs.

## 5 Conclusion

In this study, we delineated the genomic landscape of TCCA and identified TCCA-specific genomic alterations. TCCAs display more aggressive and invasive clinical features than TCAs, particularly in cases with *RET* fusions, and *BRAF^V600E^* coexisting with other point mutations. Integrating molecular and cytological testing improves the predictive value of FNAB and reduces the need for secondary surgery. Overall, targeted molecular testing can support subtype diagnosis and inform treatment strategies for TCCA.

## Data Availability

The datasets presented in this study can be found in online repositories. Raw data have been deposited to National Center for Biotechnology Information under the BioProject number PRJNA1279436.

## Supplementary material

Supplementary Figure 1 Process for screening previous literature from public database.
