# Supplementary figures and images for: Specific genomic alterations and aggressive clinical features of sporadic thyroid carcinomas in children and adolescents: findings from an in-house cohort study
Source: Front Endocrinol (Lausanne). 2025 Aug 15;16:1603571. doi: 10.3389/fendo.2025.1603571 (PMC12394056; doi:10.3389/fendo.2025.1603571)

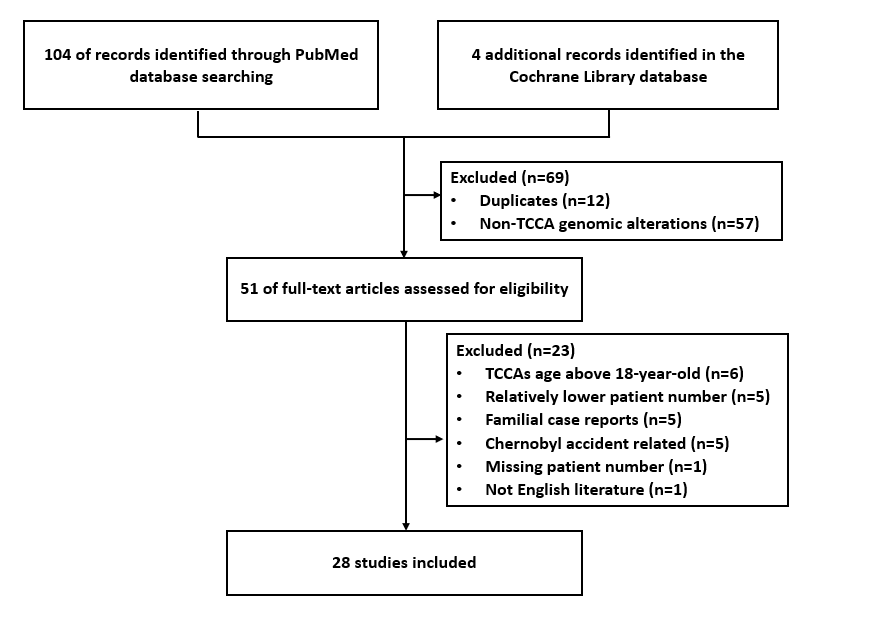

Supplement: Supplementary Figure 1 — Process for screening previous literature from public database. [file Image1.png]
