# Supplementary material for: Specific genomic alterations and aggressive clinical features of sporadic thyroid carcinomas in children and adolescents: findings from an in-house cohort study
Source: Front Endocrinol (Lausanne). 2025 Aug 15;16:1603571. doi: 10.3389/fendo.2025.1603571 (PMC12394056; doi:10.3389/fendo.2025.1603571)
Supplement: Supplementary file 2 [file Table1.docx]

**Supplementary table 1. Information of the 28 previous studies included in the analysis**

| **Author** | **Journal** |
| --- | --- |
| Mollen KP et al. | Thyroid 2022, 32(3):236-244. |
| Li Y et al. | Sci Rep 2022, 12(1):12674. |
| Clague DeHart et al. | J Clin Oncol 2022, 40(10):1081-1090. |
| Macerola E et al. | Cancers (Basel) 2021, 13(13). |
| Lee YA et al. | J Clin Invest, 2021, 131(18):e144847. |
| Potter SL et al. | Pediatric Blood & Cancer 2020, 68(1). |
| Pekova B et al. | Thyroid 2020, 30(12):1771-1780. |
| Lee YA et al. | Thyroid 2020, 30(8):1120-1131. |
| Alzahrani AS et al. | J Clin Endocrinol Metab 2020, 105(10). |
| Galuppini F et al. | Front Endocrinol (Lausanne) 2019, 10:552. |
| Wasserman JD et al. | J Clin Endocrinol Metab 2018, 103(5):2009-2015. |
| Mostoufi-Moab S et al. | Thyroid 2018, 28(1):60-67. |
| Vanden Borre P et al. | Oncologist 2017, 22(3):255-263. |
| Cordioli MI et al. | Thyroid 2017, 27(2):182-188. |
| Prasad ML et al. | Cancer 2016, 122(7):1097-1107. |
| Picarsic JL et al. | Pediatr Dev Pathol 2016, 19(2):115-122. |
| Nikita ME et al. | Thyroid 2016, 26(2):227-234. |
| Ballester LY et al. | Pediatr Dev Pathol 2016, 19(2):94-100. |
| Alzahrani AS et al. | Thyroid 2016, 26(2):235-241. |
| Henke LE et al. | Pediatr Blood Cancer 2014, 61(7):1168-1172. |
| Givens DJ et al. | Laryngoscope 2014, 124(9):E389-393. |
| Sassolas G et al. | Thyroid 2012, 22(1):17-26. |
| Monaco SE et al. | Cancer Cytopathol 2012, 120(5):342-350. |
| Stein L et al. | Thyroid 2010, 20(5):475-487. |
| Rosenbaum E et al. | Mod Pathol 2005, 18(7):898-902. |
| Penko K et al. | Thyroid 2005, 15(4):320-325. |
| Richter H et al. | Thyroid 2004, 14(12):1061-1064. |
| Kumagai A et al. | J Clin Endocrinol Metab 2004, 89(9):4280-4284. |
