# Supplementary material for: Specific genomic alterations and aggressive clinical features of sporadic thyroid carcinomas in children and adolescents: findings from an in-house cohort study
Source: Front Endocrinol (Lausanne). 2025 Aug 15;16:1603571. doi: 10.3389/fendo.2025.1603571 (PMC12394056; doi:10.3389/fendo.2025.1603571)
Supplement: Supplementary file 3 [file Table2.docx]

**Supplementary table 2. The genomic alterations and clinicopathological characteristics in TCCAs and TCAs.**

| **Patient ID** | **Group** | **Genomic Alterations** | **Age** | **Gender** | **LNM_Number** | **Tumor.Diameter.(mm)** | **Tumor.Size** | **Multifocality** | **Aspect.Radio** | **Tumor.Shape** | **Tumor.Border** | **Internal.Echo.Pattern** | **Tumor.Vascularization** | **ETE** | **LNM** |
| --- | --- | --- | --- | --- | --- | --- | --- | --- | --- | --- | --- | --- | --- | --- | --- |
| TCCA1 | TCCA | *BRAF* | 18 | M | 0 | 18 | ≤20mm | Multifocality | <1 | Regular | Clear | Nonuniform | Rare | Negative | Negative |
| TCCA10 | TCCA | *CCDC6-RET* | 16 | F | 35 | 10 | ≤20mm | Singleness | <1 | Regular | Clear | Nonuniform | Abundant | Negative | Positive |
| TCCA11 | TCCA | *PRKAR1A-RET* | 16 | F | 13 | 22 | >20mm | Multifocality | <1 | Regular | Clear | Nonuniform | Rare | Positive | Positive |
| TCA1 | TCA | *BRAF* | 47 | M | 2 | 10 | ≤20mm | Multifocality | <1 | Regular | Fuzzy | Nonuniform | Abundant | Negative | Positive |
| TCCA12 | TCCA | *EP300* | 10 | F | 7 | 20 | ≤20mm | Singleness | <1 | Regular | Clear | Nonuniform | Abundant | Negative | Positive |
| TCCA13 | TCCA | Negative | 15 | F | 6 | 35.9 | >20mm | Multifocality | <1 | Irregular | Fuzzy | Nonuniform | Abundant | Negative | Positive |
| TCA10 | TCA | *BRAF* | 75 | F | 1 | 7 | ≤20mm | Singleness | >1 | Regular | Clear | Nonuniform | Rare | Negative | Negative |
| TCA11 | TCA | *BRAF* | 36 | F | 1 | 26 | >20mm | Singleness | <1 | Regular | Clear | Nonuniform | Abundant | Negative | Negative |
| TCA12 | TCA | *BRAF* | 35 | M | 1 | 9.5 | ≤20mm | Multifocality | <1 | Regular | Fuzzy | Nonuniform | Rare | Negative | Negative |
| TCA13 | TCA | *BRAF* | 34 | F | 0 | 6 | ≤20mm | Multifocality | =1 | Regular | Fuzzy | Nonuniform | Rare | Negative | Negative |
| TCCA14 | TCCA | Negative | 15 | M | 9 | 45 | >20mm | Multifocality | <1 | Irregular | Fuzzy | Nonuniform | Abundant | Negative | Positive |
| TCA14 | TCA | *BRAF* | 33 | M | 0 | 8 | ≤20mm | Singleness | =1 | Regular | Clear | Nonuniform | Rare | Negative | Negative |
| TCCA15 | TCCA | Negative | 7 | M | 0 | 25 | >20mm | Multifocality | <1 | Irregular | Fuzzy | Nonuniform | Abundant | Positive | Negative |
| TCA15 | TCA | *BRAF* | 34 | F | 0 | 7 | ≤20mm | Singleness | <1 | Irregular | Fuzzy | Nonuniform | Rare | Negative | Negative |
| TCA16 | TCA | *BRAF* | 43 | F | 0 | 17 | ≤20mm | Singleness | >1 | Regular | Fuzzy | Nonuniform | Rare | Negative | Negative |
| TCA17 | TCA | *BRAF* | 46 | M | 7 | 9 | ≤20mm | Multifocality | <1 | Regular | Fuzzy | Nonuniform | Rare | Negative | Positive |
| TCCA16 | TCCA | *BRAF* | 18 | F | 0 | 12.1 | ≤20mm | Singleness | >1 | Irregular | Fuzzy | Nonuniform | Rare | Negative | Positive |
| TCA18 | TCA | *BRAF* | 58 | F | 2 | 8 | ≤20mm | Singleness | >1 | Regular | Fuzzy | Nonuniform | Rare | Negative | Negative |
| TCCA17 | TCCA | Negative | 17 | F | 1 | 21.5 | >20mm | Multifocality | <1 | Irregular | Clear | Nonuniform | Rare | Negative | Positive |
| TCA19 | TCA | *BRAF* | 57 | M | 0 | 6 | ≤20mm | Multifocality | =1 | Regular | Fuzzy | Nonuniform | Rare | Negative | Negative |
| TCCA18 | TCCA | *RUFY2-RET* | 10 | M | 0 | 14.5 | ≤20mm | Multifocality | >1 | Regular | Clear | Nonuniform | Rare | Negative | Negative |
| TCA2 | TCA | *BRAF* | 62 | M | 2 | 9.5 | ≤20mm | Multifocality | >1 | Irregular | Fuzzy | Nonuniform | Abundant | Negative | Positive |
| TCA20 | TCA | *BRAF* | 42 | M | 0 | 7.5 | ≤20mm | Singleness | <1 | Regular | Fuzzy | Nonuniform | Rare | Negative | Negative |
| TCA21 | TCA | *BRAF* | 65 | F | 0 | 10 | ≤20mm | Singleness | <1 | Regular | Clear | Nonuniform | Abundant | Negative | Negative |
| TCA22 | TCA | *BRAF* | 27 | M | 0 | 12 | ≤20mm | Multifocality | <1 | Irregular | Fuzzy | Nonuniform | Rare | Negative | Negative |
| TCA23 | TCA | *BRAF* | 64 | F | 1 | 22 | ≤20mm | Multifocality | <1 | Irregular | Fuzzy | Nonuniform | Rare | Negative | Positive |
| TCA24 | TCA | *BRAF* | 71 | F | 0 | 7.5 | ≤20mm | Singleness | >1 | Irregular | Clear | Nonuniform | Rare | Negative | Negative |
| TCA25 | TCA | *BRAF* | 32 | F | 1 | 14.5 | ≤20mm | Singleness | <1 | Regular | Clear | Nonuniform | Abundant | Negative | Negative |
| TCCA19 | TCCA | Negative | 18 | F | 0 | 34.8 | >20mm | Multifocality | <1 | Irregular | Fuzzy | Nonuniform | Rare | Negative | Negative |
| TCA26 | TCA | *BRAF* | 31 | F | 0 | 9 | ≤20mm | Singleness | <1 | Regular | Fuzzy | Nonuniform | Abundant | Negative | Negative |
| TCA27 | TCA | *BRAF* | 57 | F | 2 | 11 | ≤20mm | Singleness | >1 | Regular | Fuzzy | Nonuniform | Rare | Negative | Negative |
| TCA28 | TCA | *BRAF* | 58 | F | 2 | 15 | ≤20mm | Multifocality | >1 | Regular | Fuzzy | Nonuniform | Rare | Negative | Negative |
| TCA29 | TCA | *BRAF* | 30 | F | 3 | 14.5 | ≤20mm | Multifocality | <1 | Regular | Clear | Nonuniform | Rare | Negative | Negative |
| TCA3 | TCA | *BRAF* | 63 | M | 1 | 12 | ≤20mm | Multifocality | <1 | Irregular | Fuzzy | Nonuniform | Abundant | Negative | Positive |
| TCA30 | TCA | *BRAF* | 56 | F | 0 | 13 | ≤20mm | Singleness | =1 | Irregular | Clear | Nonuniform | Rare | Negative | Negative |
| TCA31 | TCA | *BRAF* | 59 | M | 0 | 8.3 | ≤20mm | Singleness | >1 | Regular | Clear | Nonuniform | Abundant | Negative | Negative |
| TCA32 | TCA | *BRAF* | 57 | F | 0 | 11 | ≤20mm | Singleness | <1 | Irregular | Clear | Nonuniform | Rare | Negative | Positive |
| TCA33 | TCA | *BRAF* | 61 | F | 1 | 7.6 | ≤20mm | Singleness | >1 | Irregular | Fuzzy | Nonuniform | Abundant | Negative | Negative |
| TCCA2 | TCCA | *BRAF* | 13 | F | 8 | 7.4 | ≤20mm | Singleness | <1 | Irregular | Clear | Nonuniform | Rare | Positive | Positive |
| TCCA20 | TCCA | Negative | 17 | F | 0 | 8.5 | ≤20mm | Singleness | <1 | Irregular | Clear | Nonuniform | Rare | Negative | Negative |
| TCA34 | TCA | *BRAF* | 47 | F | 0 | 6 | ≤20mm | Singleness | 1 | Irregular | Fuzzy | Nonuniform | Rare | Negative | Negative |
| TCA35 | TCA | *BRAF* | 48 | F | 0 | 9 | ≤20mm | Singleness | >1 | Irregular | Fuzzy | Nonuniform | Rare | Negative | Negative |
| TCA36 | TCA | *BRAF* | 53 | F | 1 | 9.5 | ≤20mm | Multifocality | <1 | Regular | Clear | Nonuniform | Rare | Negative | Negative |
| TCA37 | TCA | *BRAF* | 24 | F | 18 | 18 | ≤20mm | Singleness | <1 | Regular | Fuzzy | Nonuniform | Rare | Negative | Positive |
| TCA38 | TCA | *BRAF* | 48 | F | 2 | 6 | ≤20mm | Singleness | <1 | Regular | Fuzzy | Nonuniform | Abundant | Negative | Negative |
| TCCA3 | TCCA | *BRAF* | 14 | F | 7 | 18 | ≤20mm | Singleness | >1 | Regular | Clear | Uniform | Rare | Negative | Positive |
| TCCA4 | TCCA | *BRAF* | 15 | M | 9 | 25 | >20mm | Singleness | <1 | Irregular | Fuzzy | Nonuniform | Abundant | Negative | Positive |
| TCCA5 | TCCA | *BRAF, PTEN* | 13 | M | 10 | 19.1 | >20mm | Multifocality | <1 | Irregular | Clear | Nonuniform | Rare | Positive | Positive |
| TCA39 | TCA | *BRAF* | 42 | F | 0 | 6.1 | ≤20mm | Multifocality | >1 | Regular | Clear | Nonuniform | Rare | Negative | Negative |
| TCA4 | TCA | *BRAF* | 44 | F | 3 | 25 | >20mm | Singleness | <1 | Regular | Fuzzy | Nonuniform | Abundant | Negative | Positive |
| TCCA6 | TCCA | *BRAF, KMT2D* | 16 | F | 16 | 49 | >20mm | Multifocality | <1 | Irregular | Fuzzy | Nonuniform | Rare | Negative | Positive |
| TCCA7 | TCCA | *BRAF, ATM* | 17 | F | 11 | 35 | >20mm | Singleness | <1 | Regular | Clear | Nonuniform | Abundant | Negative | Positive |
| TCCA8 | TCCA | *BRAF, KMT2C, ZFHX3* | 17 | M | 14 | 25 | >20mm | Multifocality | >1 | Regular | Fuzzy | Nonuniform | Rare | Negative | Positive |
| TCA40 | TCA | *BRAF* | 33 | F | 0 | 9.5 | ≤20mm | Multifocality | <1 | Regular | Fuzzy | Nonuniform | Rare | Negative | Negative |
| TCCA9 | TCCA | *ARID1B, RUFY2-RET* | 17 | F | 8 | 25 | >20mm | Multifocality | <1 | Regular | Fuzzy | Nonuniform | Abundant | Positive | Positive |
| TCA5 | TCA | *BRAF* | 46 | F | 3 | 12 | ≤20mm | Singleness | <1 | Irregular | Fuzzy | Nonuniform | Abundant | Negative | Positive |
| TCA6 | TCA | *BRAF, KMT2A* | 50 | F | 2 | 29 | >20mm | Multifocality | >1 | Irregular | Fuzzy | Nonuniform | Rare | Positive | Positive |
| TCA7 | TCA | *BRAF, DNMT3A* | 54 | F | 1 | 15 | ≤20mm | Singleness | >1 | Irregular | Clear | Nonuniform | Abundant | Negative | Positive |
| TCA8 | TCA | Negative | 44 | F | 0 | 6 | ≤20mm | Singleness | >1 | Irregular | Clear | Nonuniform | Abundant | Positive | Negative |
| TCA9 | TCA | Negative | 34 | F | 0 | 8 | ≤20mm | Multifocality | <1 | Regular | Fuzzy | Nonuniform | Rare | Negative | Negative |
